# Promotion of Viral IRES-Mediated Translation Initiation under Mild Hypothermia

**DOI:** 10.1371/journal.pone.0126174

**Published:** 2015-05-07

**Authors:** Maria Licursi, Ricardo A. Carmona-Martinez, Seyd Razavi, Kensuke Hirasawa

**Affiliations:** Division of BioMedical Science, Faculty of Medicine, Memorial University of Newfoundland, 300 Prince Philip Dr., St. John’s, NL, A1B 3V6, Canada; University of British Columbia, CANADA

## Abstract

Internal ribosome entry site (IRES)-mediated translation is an essential replication step for certain viruses. As IRES-mediated translation is regulated differently from cap-dependent translation under various cellular conditions, we sought to investigate whether temperature influences efficiency of viral IRES-mediated translation initiation by using bicistronic reporter constructs containing an IRES element of encephalomyocarditis virus (EMCV), foot-and-mouth disease virus (FMDV), hepatitis C virus (HCV), human rhinovirus (HRV) or poliovirus (PV). Under mild hypothermic conditions (30 and 35°C), we observed increases in the efficiency of translation initiation by HCV and HRV IRES elements compared to translation initiation at 37°C. The promotion of HRV IRES activity was observed as early as 2 hours after exposure to mild hypothermia. We also confirmed the promotion of translation initiation by HRV IRES under mild hypothermia in multiple cell lines. The expression levels and locations of polypyrimidine tract-binding protein (PTB) and upstream of N-Ras (unr), the IRES trans-acting factors (ITAFs) of HCV and HRV IRES elements, were not modulated by the temperature shift from 37°C to 30°C. Taken together, this study demonstrates that efficiency of translation initiation by some viral IRES elements is temperature dependent.

## Introduction

Translation of eukaryotic mRNAs is comprised of four stages: initiation, elongation, termination and ribosome recycling. Initiation is the most tightly regulated step, and can cause rapid changes in gene expression in various intracellular and extracellular environments [1–3]. The majority of eukaryotic mRNA translation is initiated by a mechanism known as cap-dependent translation, termed as a cap structure at the terminal base of the 5’ untranslated region (5’ UTR) is required [4]. Conversely, translation of some viral and cellular mRNAs can be initiated by the direct binding of a ribosome to a unique RNA element called an internal ribosome entry site (IRES) [5–8]. IRES elements were first discovered in the mRNAs of the *Picornaviridae* [6,9]. Since then, several other viral and cellular mRNAs have been reported to contain an IRES element [5,10–14]. The IRES contains a high degree of RNA secondary structure and recruits the 40S ribosomal subunit in a cap-independent manner. The initiation codon is in close proximity or, in some cases, is reached by scanning. In addition to canonical initiation factors, cellular proteins known as IRES trans-acting factors (ITAFs) are required for efficient IRES-mediated translation [15,16]. The ITAFs required for translation initiation are unique to individual IRES elements [15], considering this, cellular expression levels of ITAFs are known to influence tissue tropism of viruses [17].

Under cellular stress, cap-dependent protein synthesis is impaired while translation driven by IRES elements is often maintained or even upregulated [18–20]. This feature of IRES-mediated translation is advantageous for viral RNA to be translated efficiently during viral replication. Moreover, cellular mRNAs containing IRES elements play critical roles in maintaining cellular homeostasis or inducing apoptosis during cellular stress [21,22]. Although there have been a significant number of studies on translation initiation by cellular IRES elements under stress conditions [23–26], less is known about regulation of viral IRES-mediated translation by cellular stresses. We previously clarified that amino acid starvation increases efficiency of translation initiation of encephalomyocarditis virus (EMCV) and foot and mouth disease virus (FMDV) IRES elements by dephosphorylation of eukaryotic translation initiation factor 4E-binding protein (4E-BP) [27]. In this study, we systematically investigated the effect of temperature on efficiency of translation initiation by viral IRES elements using bicistronic IRES reporter constructs.

## Results

**Fig 1:**
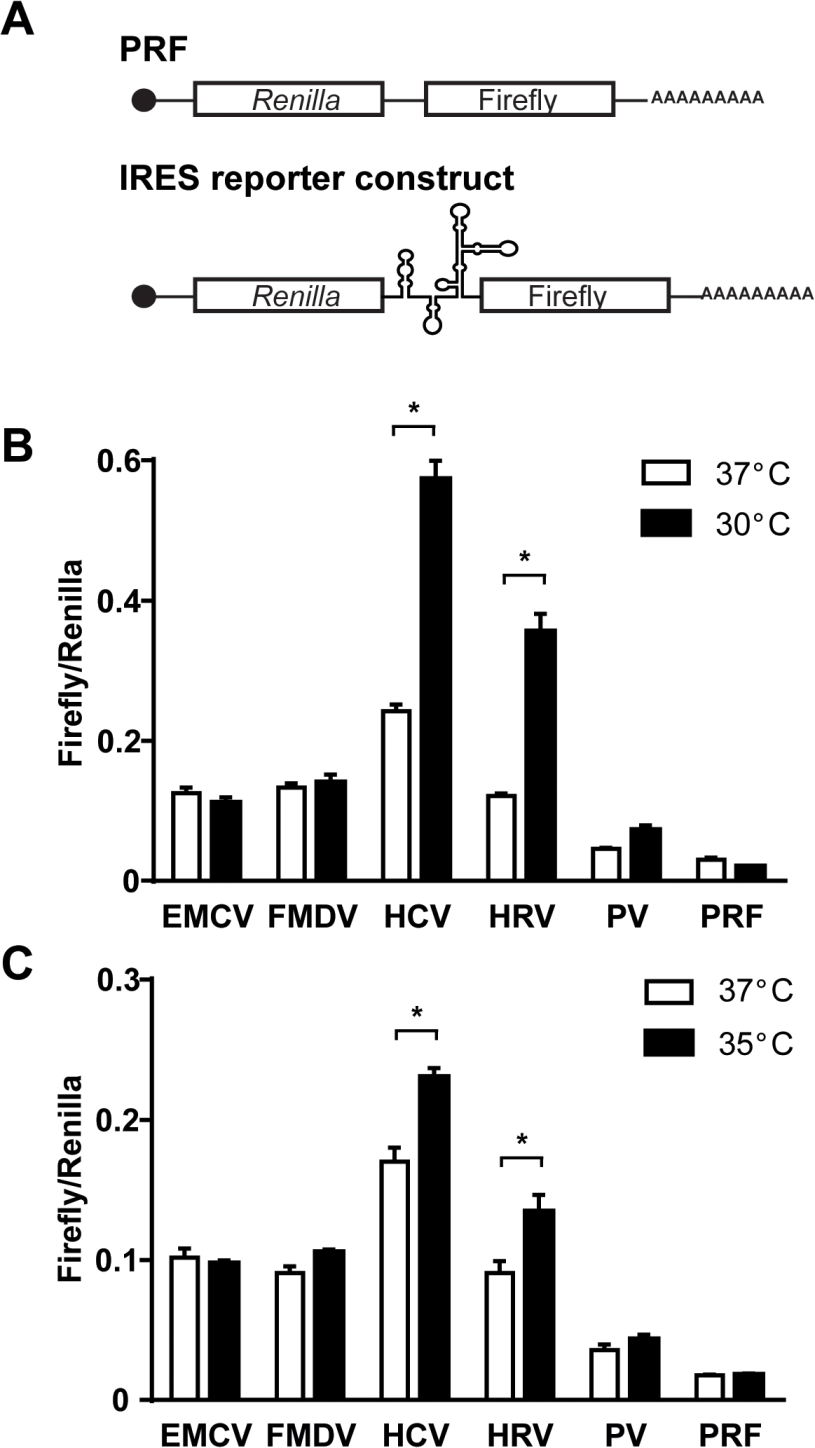
Temperature-dependent translation initiation by viral IRES elements. **(A)** Schematic representation of the bicistronic reporter constructs. NIH3T3 cells were transfected with bicistronic reporter constructs containing an IRES element from EMCV, FMDV, HCV, HRV or PV, or a control pRF construct. 24 hours post-transfection, cells were cultured at 30°C **(B)**, 35°C **(C)**, or 37°C for 8 hours. Firefly and *Renilla* luciferase units were measured using the Dual-Luciferase Reporter Assay System. Firefly/Renilla represents the ratio of viral IRES-mediated translation to cap-dependent translation. Data are expressed as the mean ± SE of 3 independent samples. Statistical analysis was conducted using two-way ANOVA and multiple comparison test. *p<0.01.

**Fig 2:**
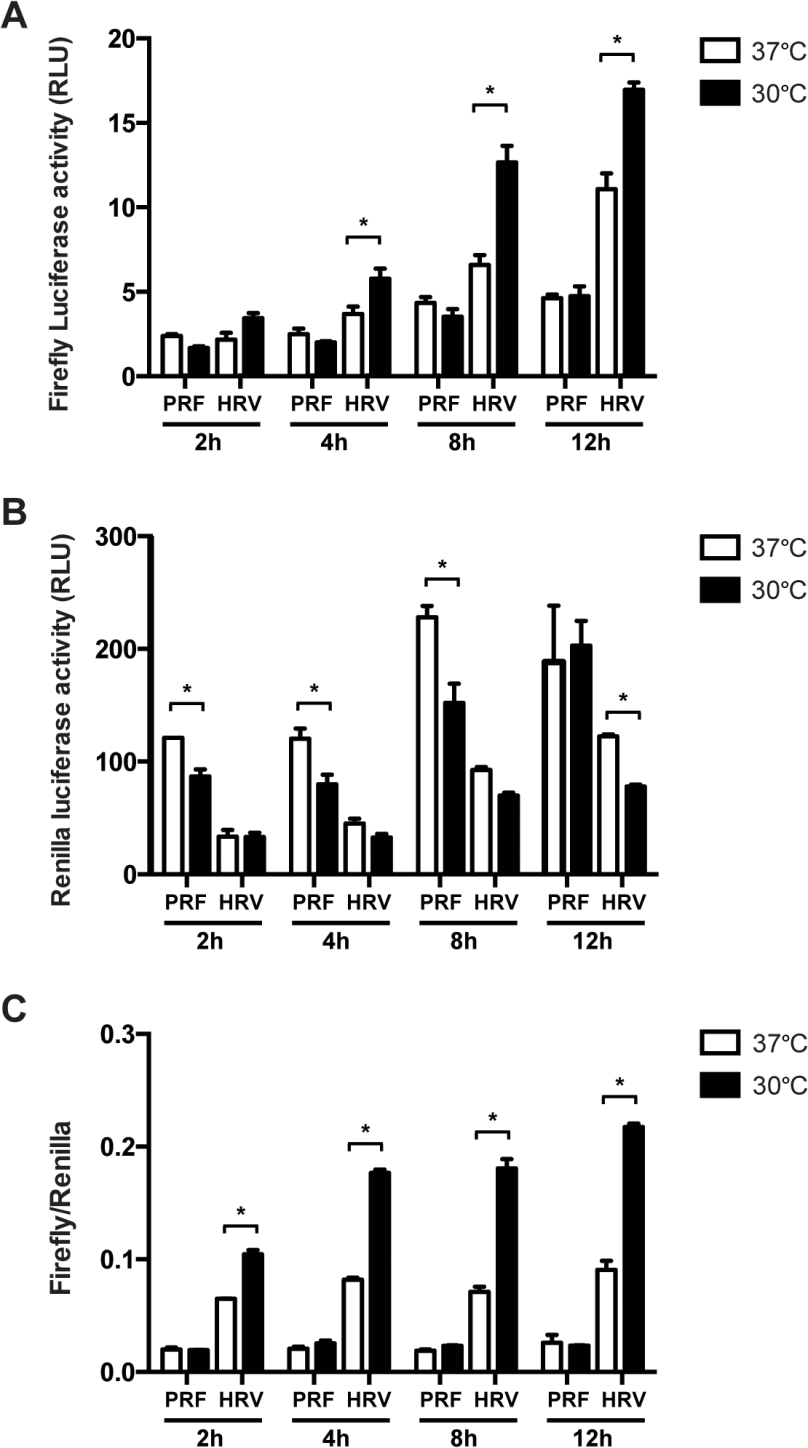
Time course of the effect of mild hypothermia on promotion of HRV IRES-mediated translation. NIH3T3 cells transfected with the pRF or HRV IRES reporter constructs were exposed to mild hypothermia (30°C) for 2, 4, 8 and 12 hours. Absolute values of firefly luciferase **(A)**, absolute values of *Renilla* luciferase **(B)** and ratio of Firefly/Renilla luciferase were measured using the Dual-Luciferase Reporter Assay System. Data are expressed as the mean ± SE of 3 independent samples. Statistical analysis was conducted using two-way ANOVA and multiple comparison test. *p<0.01.

To determine whether culture temperature influences viral IRES-mediated translation NIH3T3 cells were transfected with bicistronic IRES reporter constructs (encephalomyocarditis virus (EMCV), foot and mouth disease virus (FMDV), human rhinovirus (HRV), hepatitis C virus (HCV), poliovirus (PV) or the empty vector (pRF)) (Fig 1A). The reporter construct generates a single mRNA containing a viral IRES element between *Renilla* and firefly luciferase sequences in transfected cells. The viral IRES element initiates translation of firefly luciferase. *Renilla* luciferase activity, which serves as control, represents cap-dependent translation since its translation is initiated by the cap structure at the 5’ UTR of the mRNA. The efficiency of viral IRES-mediated translation was evaluated by the ratio of firefly (viral IRES-mediated translation) to *Renilla* (cap-dependent translation) luciferase activity. Cells were incubated at the different temperatures (30, 35 and 37°C) for 8 hours. HCV and HRV IRES-mediated translation at 30°C was significantly promoted compared to 37°C, while translation initiation by the other IRES elements remained unchanged (Fig 1B). Similar promotion of the IRES-mediated translation was observed at 35°C (Fig 1C). We further confirmed the promotion of HRV IRES activity under mild hypothermia at various time points (2, 4, 8 and 12 hours) (Fig 2). The absolute values of firefly luciferase activity were increased in cells transfected with HRV IRES reporter construct exposed to mild hypothermia for 4, 8 and 12 hours relative to cells cultured at 37°C (Fig 2A). In contrast, *Renilla* luciferase activity of HRV reporter construct was decreased only at 12 hours after exposure to mild hypothermia (Fig 2B). As a result, when HRV IRES-mediated translation (firefly luciferase) was standardized to cap-dependent translation (*Renilla* luciferase), we found that HRV IRES activity was significantly increased at 30°C compared to 37°C (Fig 2C). These results clearly demonstrate that translation initiation by viral IRES elements is temperature dependent.

**Fig 3:**
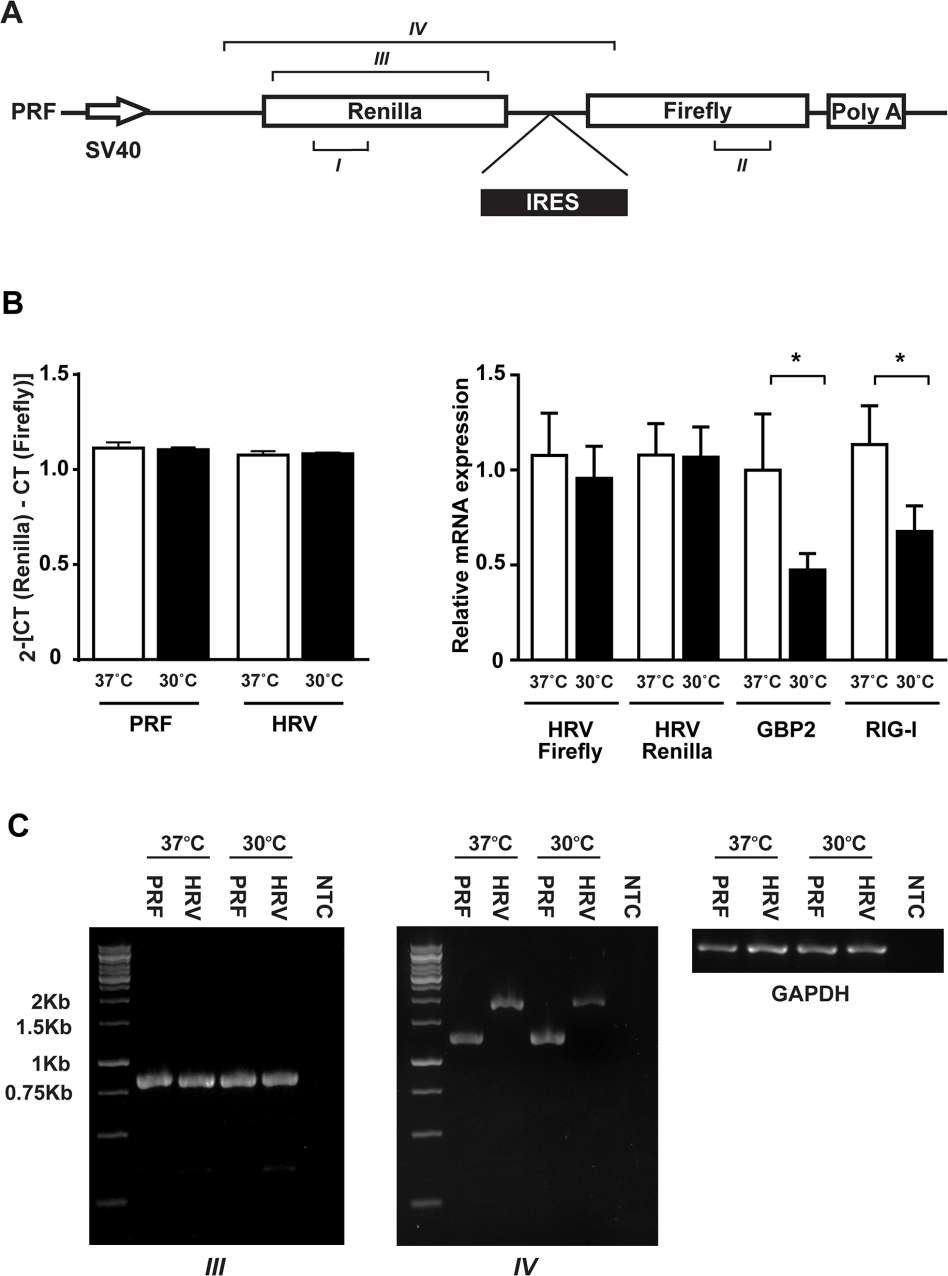
Absence of modifications of reporter construct mRNAs in response to the temperature shift. **(A**) Schematic representation of the bicistronic reporter construct. Positions of the different regions amplified by qRT-PCR (primer region I and II) or by RT-PCR (amplicons III and IV) are indicated. **(B)** NIH3T3 cells were transfected with HRV or control pRF reporter construct for 24 hours and, then, incubated at 37°C or 30°C for 8 hours. qRT-PCR analysis of *Renilla* (primer region I) and firefly luciferase (primer region II) mRNA of PRF and HRV IRES reporter construct (left) and of HRV firefly luciferase, HRV *Renilla* luciferase, GBP2 and RIG-I mRNA (right). The expression levels were normalized to GAPDH and reported as comparisons to the expression level at 37°C. The *Renilla* luciferase/firefly luciferase ratio was calculated as 2-[CT (Renilla)—CT (Firefly)]. Data are expressed as the mean ± SE of 3 independent samples. Statistical analyses were performed using a t-test. *p<0.01. **(C)** RT-PCR analysis for amplicon III, amplicon IV and GAPDH.

To confirm that the temperature dependence occurs at the translational level, but not at the transcriptional level, we conducted gene expression analysis on the reporter construct mRNAs (Fig 3). When the expression levels of *Renilla* luciferase (primer region I) and firefly luciferase (primer region II) mRNA were examined by quantitative RT-PCR, no difference in the ratio of *Renilla*/firefly luciferase mRNA was observed between 30°C and 37°C (Fig 3B). This indicates that the temperature shift does not induce splicing of the reporter mRNA or generate a cryptic promoter, which could lead to changes in the ratios of firefly/*Renilla* luciferase activity. Alternatively, the presence of an IRES element in mRNA could promote its stability at low culture temperatures [28]. To test this, we determined if HRV IRES element regulates the stability of the construct mRNA (Fig 3B). The amount of HRV construct mRNA was not changed between 30°C and 37°C while transcription of endogenous mRNAs such as guanylate binding protein 2 (GBP2) and retinoic acid-inducible gene 1 (RIG-I) was reduced at 30°C. This is most likely because the SV40 promoter on the reporter construct is less sensitive to the temperature shift compared to endogenous promoters. Nevertheless, the HRV IRES element did not increase the amount of the reporter construct mRNA at 30°C. To further eliminate the possibility that mild hypothermia induces alternative spicing of the HRV IRES reporter construct, we conducted RT-PCR analysis targeting two regions of reporter mRNA (amplicon III and IV) (Fig 3C). We did not find multiple transcripts, demonstrating that alternative splicing was not induced by the temperature shift. Taken together, the promotion of viral IRES-mediated translation under mild hypothermia is induced at the translational level but not at the transcriptional level.

**Fig 4:**
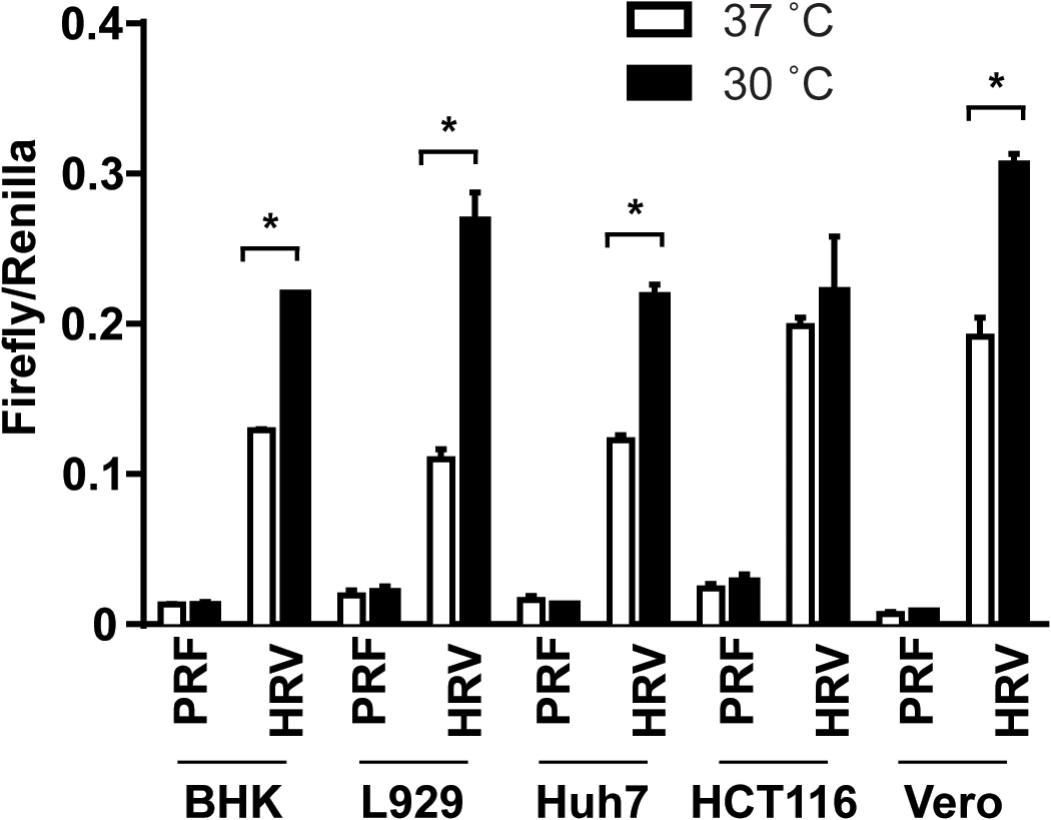
Temperature sensitivity of HRV IRES-mediated translation in different cell types. BHK, L929, Huh7 HCT116 and Vero cells were transfected with the HRV IRES construct or a control pRF reporter construct, 24 hours later transfected cells were incubated at 37°C or 30°C for 8 hours. Firefly and *Renilla* luciferase units were measured using the Dual-Luciferase Reporter Assay System. Firefly/Renilla represents the ratio of viral IRES-mediated translation to cap-dependent translation. Data are expressed as the mean ± SE of 3 independent samples. Statistical analysis was conducted using two-way ANOVA and multiple comparison test. *p<0.01.

We next determined if the temperature-dependent IRES-mediated translation is a cell-type specific phenomenon (Fig 4). Hamster kidney fibroblast cells (BHK), mouse fibrosarcoma cells (L929), human hepatoma cells (Huh7), human colorectal carcinoma cells (HCT116) and monkey kidney epithelium cells (Vero) were transfected with HRV IRES or control pRF reporter constructs and then incubated at 30°C or 37°C for 8 hours. Similar to the results observed using NIH3T3 cells (Fig 1), we found that efficiency of translation initiation by HRV IRES element was significantly higher at 30°C than at 37°C in all the cell lines tested with the exception of HCT116. This suggests that the cellular machinery responsible for the temperature dependent IRES-mediated translation is generally conserved among these cell lines with different origins.

**Fig 5:**
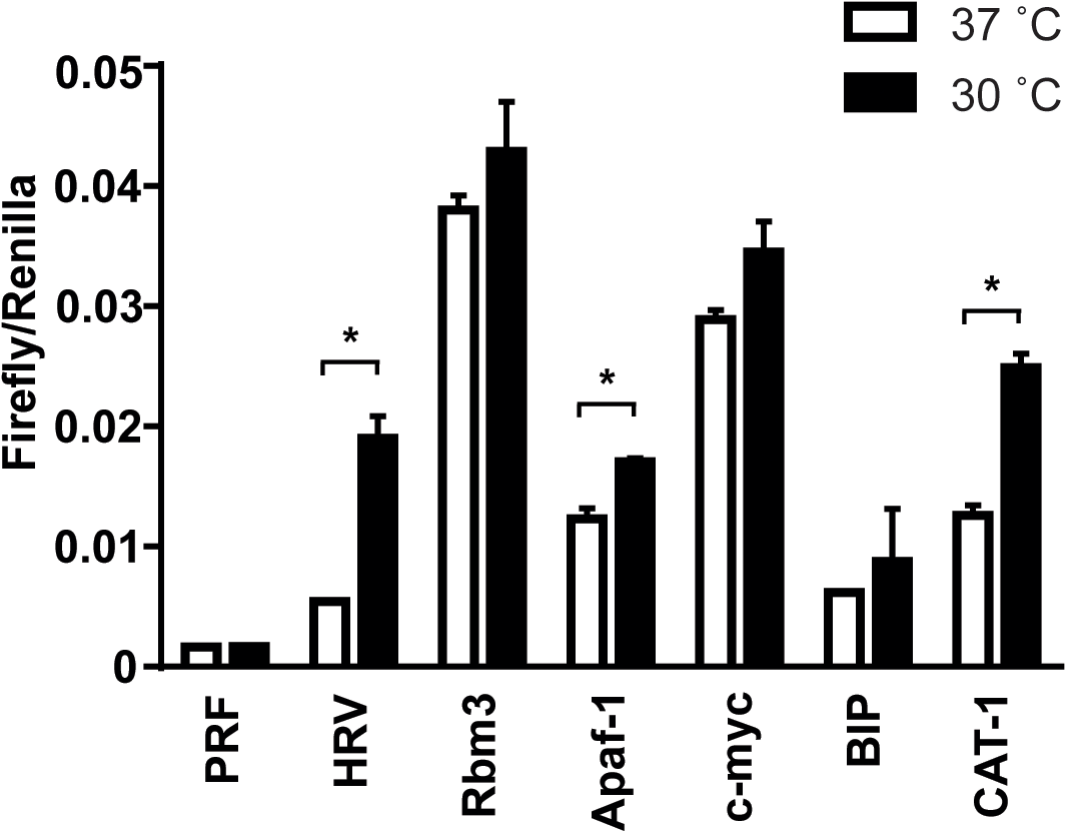
Temperature sensitivity of cellular IRES-mediated translation. NIH3T3 cells were transfected with a HRV, Rbm3, Apaf-1, c-myc, BIP, CAT-1 construct or a control pRF reporter construct and 24 hours later were incubated at 37°C (white bar) or 30°C (black bar) for 8 hours. Firefly and *Renilla* luciferase units were measured using the Dual-Luciferase Reporter Assay System. Firefly/Renilla represents the ratio of viral IRES-mediated translation to cap-dependent translation. Data are expressed as the mean ± SE of 3 independent samples. Statistical analysis was conducted using two-way ANOVA and multiple comparison test. *p<0.01.

To determine if the temperature dependent translation initiation by HCV and HRV IRES elements can also be observed in cellular IRES elements, we utilized bicistronic reporter constructs containing a cellular IRES element of RNA-binding motif protein 3 (Rbm3), human apoptotic protease-activating factor-1 (Apaf-1), immunoglobulin heavy chain binding protein (BIP), c-myc or rat cationic amino-acid (arginine/lysine) transporter-1 (CAT-1). Among the cellular IRES elements, we found that Apaf-1 and CAT-1 IRES translation initiation efficiency was increased at 30°C compared to 37°C, although the degree of the increase was less than that of the HRV IRES element (Fig 5). This indicates that mild hypothermia also promotes translation initiation of certain cellular IRES elements.

**Fig 6:**
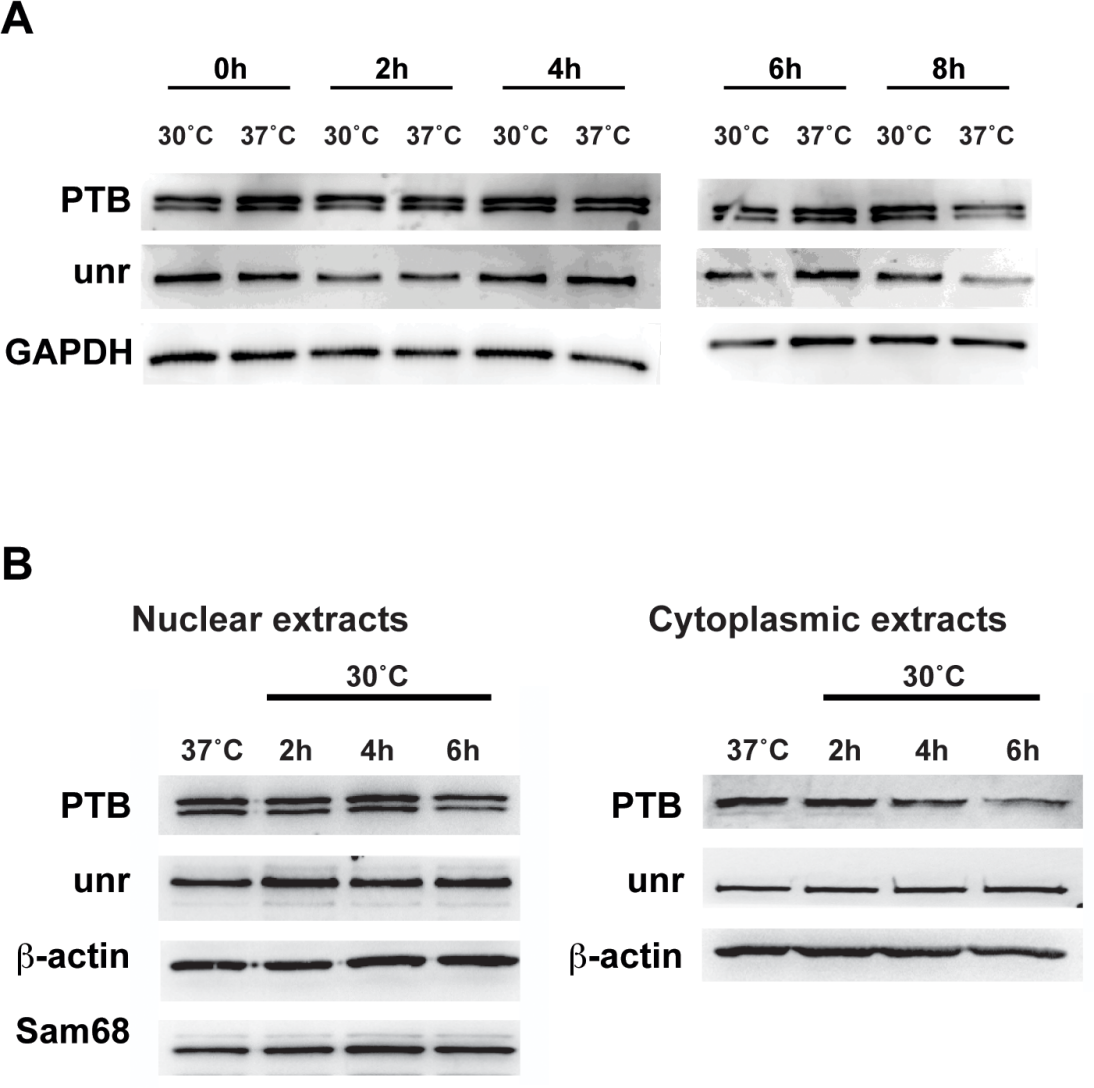
Expression (A) and cellular localization (B) of PTB and unr under 30°C and 37°C culture. NIH3T3 cells were cultured at 30°C and 37°C for 0, 2, 4, and 6 hours. Western blot analysis was performed using antibodies against PTB, unr, β-actin and Sam68.

Next, we sought to identify cellular mechanisms responsible for the promotion of viral IRES-mediated translation under mild hypothermia. IRES-transacting factors (ITAFs), which are unique to individual IRES elements, are one of major factors known to regulate IRES-mediated translation. Therefore, we hypothesized that translation initiation temperature dependency can be caused by different expression levels of the ITAFs or their localization within cellular compartments. To test this possibility, we first examined if expression levels of polypyrimidine tract binding protein (PTB) and upstream of N-Ras (unr), known to bind to HRV and HCV IRES elements, were modified under mild hypothermia (Fig 6A). Western blot analysis using antibodies against PTB and unr revealed that the expression levels are similar between 30°C and 37°C. We further compared the cellular localization of PTB and unr between at 30°C and at 37°C by western blot analysis of nuclear and cytoplasmic extracts (Fig 6B). Furthermore, we did not observe the translocation of PTB and unr into the cytoplasm from the nucleus at 2, 4 and 6 hours after mild hypothermia treatment.

## Discussion

Thirty-three degrees Celsius is considered to be the optimal growth temperature for certain variants of HRV [29,30]. The HRV IRES element is involved in the temperature sensitivity of HRV replication as evident by the ability of the chimeric poliovirus carrying the HRV2 IRES element that replicate with greater efficiency at 33°C than at 37°C in non-neuronal cell lines [31]. Among cellular IRES elements, Rbm3 was reported to initiate its translation more efficiently when cells were cultured at 33°C compared to at 37°C [25]. The literature to date clearly demonstrates an influence of temperature on IRES-dependent translation initiation. In our study, we conducted a systemic screening of temperature sensitivities of IRES-dependent translation initiation by five viral IRES constructs. We found that mild hypothermia (30°C and 35°C) promotes translation initiation by HRV and HCV IRES elements.

A potential major pitfall of this work was that the changes of culture temperature could modulate the bicistronic reporter system at the transcriptional level, which may lead to the misinterpretation of IRES-mediated translation. One possibility is that mild hypothermia induces aberrant transcripts processed either by a cryptic promoter or by RNA splicing [32]. Additionally, it is known that a lower culture temperature (32°C) increases the stability of mRNA containing Cold-inducible RNA binding protein (CIRP) IRES element [28]. As demonstrated by the qRT-PCR analysis (Fig 3B), the change of temperature did not affect the ratio of firefly and *Renilla* luciferase transcripts, excluding the possible activation of a cryptic promoter under mild hypothermia. In addition, mild hypothermia did not increase amount of the HRV reporter construct mRNA (Fig 3B), suggesting that the HRV IRES element, unlike the CIRP IRES element, does not increase stability of its own mRNAs under mild hypothermia. Finally, RT-PCR analysis demonstrated that mild hypothermia does not induce RNA splicing (Fig 3C), which may manipulate our findings. The preceding analyses confirmed that the promotion of IRES activity is induced at the translational level, but not at the transcriptional level.

Chappell et al. reported that mild hypothermia (33°C culture for 24 hours) promotes translation initiation by the Rbm3 IRES element in NIH3T3 cells [25]. The promotion of Rbm3 IRES-dependent translation initiation was not observed in NIH3T3 cells when they were transfected with the Rbm3 reporter construct and cultured at 30°C for 8 hours. We believe that the discrepancy between the temperature and length of the incubation period in these two studies may have contributed to the contrasting results.

Considering IRES elements require different ITAFs for efficient translation initiation [15], the promotion of IRES-mediated translation under mild hypothermia may be dependent on the status of the ITAFs involved in the translation initiation. PTB is a well-known ITAF required for translation initiation by HRV. Its role in HCV translation initiation is controversial, while some studies suggest that PTB binding enhances HCV-mediated translation [33,34], other reports indicate an inhibitory effect [35]. Conversely, unr has been shown to promote HRV IRES-mediated translation [36]. Although unr also binds the HCV IRES [37], no functional analysis has yet reported its effect on HCV-mediated translation. unr is a member of the cold-shock protein family, and its expression can be upregulated in response to cold-shock or nutrient stress [38–40]. Separately, PTB translocates from the nucleus to the cytoplasm under stress conditions such as viral infection and/or cell death [41,42]. As shown in Fig 6A, mild hypothermia did not modulate the expression levels of unr and PTB. Moreover, we did not observe the translocation of unr or PTB from the nucleus to the cytoplasm under mild hypothermia either (Fig 6B). Another possibility that remains to be studied is that mild hypothermia regulates posttranslational modification of the ITAFs, which may affect its ability to bind the IRES elements [43,44].

## Materials and Methods

### Cells and reagents

NIH3T3, BHK, L929, HCT116, Huh7 and Vero cells were obtained from the American Type Culture Collection. All cell lines used in this study were maintained in high glucose Dulbecco's modified Eagle's medium (DMEM) (Invitrogen) with 10% fetal bovine serum (FBS) (Cansera). Antibodies against PTB (hnRNP1-N20), UNR (G-20) and Sam68 (S-331) were obtained from Santa Cruz Biotechnology, and GAPDH from Abcam. Antibody against β-actin was obtained from Sigma.

### Plasmids

The following IRES elements were used in this study: encephalomyocarditis virus (EMCV) [45], foot and mouth disease virus (FMDV) [46], hepatitis C virus (HCV) [46], human rhino virus (HRV) [5], RNA-binding motif protein 3 (Rbm3) [47], human apoptotic protease-activating factor-1 (Apaf-1) [48], immunoglobulin heavy chain binding protein (BIP) [49], c-myc [49] or rat cationic amino-acid (arginine/lysine) transporter-1 (CAT-1) [50]. The bicistronic reporter constructs were prepared by inserting an IRES element into the bicistronic pRF vector as described previously [51].

### Luciferase assay

Cells were washed with PBS and lysed with Passive Lysis Buffer (Promega). *Renilla* luciferase and firefly luciferase activities were measure using the Dual-Luciferase Reporter Assay System (Promega). Cell lysates (10μl) were mixed with luciferase assay reagent (50μl) and luciferase activity was measured as relative light units (RLU) in a Fluoroskan Ascent luminometer (Labsystems) for 10 seconds.

### qRT-PCR and RT-PCR

RNA was isolated from cells using TRIzol (Invitrogen) according to the manufacturer’s instructions. To remove plasmid DNA, isolated RNA was treated with Turbo DNA-free DNase (Ambion) according to the manufacturer’s instructions. RNA (0.5μg) was reverse transcribed (RT) to cDNA from random hexamers using the first-strand cDNA synthesis kit from Amersham Biosciences. Quantitative RT-PCR (qRT-PCR) was performed using firefly luciferase and *Renilla* luciferase primers described by Holcik M. et al. [52], and GBP2, RIG-I and GAPDH primers described by Komatsu Y et al. [53]. Primers were validated using a 5-point, 5-fold dilution series of pRF plasmid spiked into RNA isolated from untransfected NIH3T3 cells. The absence of non-specific amplification was confirmed by observing a single peak in the melt-curve analysis, confirmation of the expected amplicon size by agarose gel analysis and the absence of amplification in the no template control wells. qPCR was then performed in triplicate on the StepOnePlus (Applied Biosystems) using Power SYBR Green PCR Master Mix (Applied Biosystems). Cycling conditions were: 95°C for 10 min, 40 cycles at 95°C for 15 s and 60°C for 1 min followed by melt-curve analysis. Primers used for RT-PCR analysis of amplicon III: the Forward 5’-gataactggtccgcagtggt-3’ and Reverse 5’-actcgctcaacgaacgattt-3’; and for amplicon IV: Forward 5’-gggcttgtcgagacagagaa-3’ and Reverse P2R 5’-tctcttcatagccttatgcagttgc-3’ described by Van Eden et al. [54]. Cycling conditions were: 98°C for 1 min, 40 cycles at 98°C for 15 sec, 63°C for 30 sec and 72°C for 1 min; followed by a final extension of 10 min at 72°C.

### Western Blot analysis

Cells were washed with PBS and lysed with RIPA buffer containing 0.1% SDS, 10 μg of aprotinin ml^-1^, 100 μg of PMSF ml^-1^ and 1% phosphatase inhibitor cocktail (Sigma). The samples were subjected to SDS-PAGE, and transferred to nitrocellulose membranes (Bio-Rad). The membrane was blocked with 5% skim milk in TBS containing 0.1% Tween 20 and then incubated with the primary antibody, followed by secondary antibody (peroxidase-conjugated anti-rabbit, anti-goat or anti-mouse) (Santa Cruz Biotechnology). Specific bands were detected using Inmobilon Western (Milipore). Nuclear and cytoplasmic extracts were obtained using the Nuclear Extract Kit (Active Motif).
